# Shift work, work time control, and informal caregiving as risk factors for sleep disturbances in an ageing municipal workforce

**DOI:** 10.5271/sjweh.3937

**Published:** 2021-03-31

**Authors:** Marianna Virtanen, Saana Myllyntausta, Jenni Ervasti, Tuula Oksanen, Paula Salo, Jaana Pentti, Mika Kivimäki, Annina Ropponen, Jaana I Halonen, Jussi Vahtera, Sari Stenholm

**Affiliations:** School of Educational Sciences and Psychology, University of Eastern Finland, Joensuu, Finland; Department of Clinical Neuroscience, Division of Insurance Medicine, Karolinska Institutet, Stockholm, Sweden; Department of Public Health, University of Turku and Turku University Hospital, Turku, Finland; Centre for Population Health Research, University of Turku and Turku University Hospital; Turku, Finland; Finnish Institute of Occupational Health, Helsinki, Finland; Institute of Public Health and Clinical Nutrition, University of Eastern Finland, Kuopio, Finland; Department of Psychology, University of Turku, Turku, Finland; Clinicum, Faculty of Medicine, University of Helsinki, Finland; Department of Epidemiology and Public Health, University College London, London, UK; Department of Health Security, Finnish Institute for Health and Welfare, Helsinki, Finland

**Keywords:** insomnia, municipal employe, older employee, shift worker, working hour

## Abstract

**Objectives:**

This study aimed to examine the contribution of shift work, work time control (WTC) and informal caregiving, separately and in combination, to sleep disturbances in ageing employees.

**Methods:**

Survey data were obtained from two prospective cohort studies with repeated measurements of working conditions, informal caregiving, and sleep disturbances. We used fixed-effect conditional logistic regression analysis to examine whether within-individual changes in shift work, WTC and informal caregiving were associated with changes in sleep. Secondary analyses included between-individuals comparison using standard logistic regression models. Results from the two cohorts were pooled using meta-analysis.

**Results:**

Low WTC and informal caregiving were associated with sleep disturbances in within-individual analyses [odds ratios (OR) ranging between 1.13 (95% confidence interval 1.01–1.27) and 1.48 (95% CI 1.29–1.68)] and in between-individuals analyses [OR 1.14 (95% CI 1.03–1.26) to 1.33 (1.19–1.49)]. Shift work alone was not associated with sleep disturbances, but accumulated exposure to shift work, low WTC and informal caregiving was associated with higher risk of sleep disturbances (OR range 1.21–1.76). For some of the sleep outcomes, informal caregiving was related to a higher risk of sleep disturbances when WTC was low and a lower risk when WTC was high.

**Conclusions:**

Informal caregiving and low WTC are associated with risk of sleep disturbances among ageing employees. The findings also suggest that low WTC in combination with informal caregiving may increase the risk of sleep disturbances whereas high WTC may alleviate the adverse impact of informal caregiving on sleep.

Sleep disturbances are common among working populations (1), and ageing is associated with many negative changes in sleep, such as more frequent arousals, shorter sleep duration and decreased slow-wave sleep (2, 3). Sleep disturbances increase the risk of various health problems, including the metabolic syndrome, type 2 diabetes, and premature mortality (4–6). Among the employed, sleep disturbances and insufficient sleep have also been associated with impaired work performance, occupational injuries, absenteeism, work disability, and increased healthcare costs (6–8). The high prevalence of sleep disturbances and the related burden on the individual, the workplace, and the healthcare system underlines the need to find new targets for prevention.

Time-related factors that may make life irregular or hectic have gained increasing attention in studies of factors that may contribute to sleep disturbances. These include, for example, shift work (ie, work schedule including irregular or unusual working hours, even during night) and informal caregiving (ie, caring for ill or disabled child, spouse or other member of a person’s social network). Shift work can disrupt the normal sleep-wake cycle and may increase the risk of health problems due to sleep deprivation, impaired sleep and fatigue (9, 10). Furthermore, the prevalence of informal caregiving increases with age and research has shown associations of informal caregiving with sickness absence (11) and cardiovascular diseases (12), which may be partially mediated through high levels of stress and sleep disturbances (13–16). However, the studies on the association between informal caregiving and sleep have rarely focused on participants in employment and their working conditions (16, 17) although, eg, in the Europe and US, more than 50% of caregivers are employed (18, 19). The few longitudinal studies on the association between informal caregiving and sleep disturbances among employees have produced inconsistent findings (20, 21).

In addition, it is unclear whether having influence on working hours at the workplace, that is, work time control (WTC), might alleviate the risk of sleep disturbances among ageing employees. WTC refers to, for example opportunities to self-determine the length of the workday, the start and end times of a duty period, taking of breaks, running of private errands during work, and the scheduling of vacations and other type of leave from work (22–24). WTC is thus flexibility based on employees’ rather than employers’ needs and the positive effect on well-being has been assumed to result from greater possibilities to balance one’s resources and time-use to better cope with job- and home-based demands. There are at least three factors underlying the positive effects of employee-oriented flexibility in working life: (i) sustainable healthy work obtained by increased opportunities for employees to recover from demands at work; (ii) improved balance between work and family life in a changing working life; and (iii) benefits for the organizations in terms of attaining and maintaining a committed, skilled and healthy workforce (24). Low WTC has previously been associated with sleep disturbances (22, 25–27), depressive symptoms (27–29), work accidents (30) and sickness absence (23), and high WTC has been shown to be associated with extended working life among older employees (31). WTC may therefore be one of the modifiable factors that protect older employees from sleep disturbances.

In the present study, we utilized data from two prospective cohort studies to examine whether shift work, low WTC and informal caregiving, separately or in combination, are associated with increased risk of sleep disturbances among older employees. To minimize confounding by between-individual differences, we used repeated measurements of shift work, WTC, and informal caregiving and applied the fixed-effect method (within-individual analysis), which uses individuals as their own controls, thus controlling for all time-invariant measured and unmeasured confounders.

## Methods

### Study populations

The participants were from the Finnish Public Sector (FPS) study (32) and the Finnish Retirement and Ageing (FIREA) study (33), ongoing cohort studies of municipal employees. In FPS, we used survey responses from waves 2012, 2014 and 2016. We restricted the data to those aged ≥50 who had responded to a minimum of two surveys (N=24 418) to be eligible for the fixed-effect (within-individual) analysis (see supplementary material, www.sjweh.fi/show_abstract.php?abstract_id=3937, for the final number of participants in each fixed-effect analysis). For a secondary analysis examining onset of sleep disturbances with repeated data (a between-individuals approach), we used the data of those participants who had survey data of two subsequent time points (2012–14 or 2014–16 or both; N=23 613). For this analysis, we restricted the data to those without sleep disturbances at the baseline survey (N=17 615).

The FIREA study cohort consists of municipal employees whose personal statutory retirement date (ie, individual old age pension date) was between 2014–2019. The first survey was sent to the eligible population 18 months before the statutory retirement date. In Finland, the public sector employees’ retirement ages are regulated by the Public Sector Pensions Act and the personal statutory age is calculated by the Pension Institute for Public Sector Employees (Keva). Thereafter, annual questionnaires have been sent to the respondents, and the present data include up to six surveys collected between 2013–2019. We included all study waves during which the participant was still employed and excluded study waves after the participant retired. The participants who were employed on at least two survey points (N=2838) formed the analytical sample for the fixed effect (within-individual) analysis. For between-individuals analysis, we used the data of those participants who had survey data of two subsequent time points (N=2771). We restricted the data to those without sleep disturbances measured at baseline (N=2084).

The FPS and FIREA studies were conducted according to the Declaration of Helsinki and approved by the Ethics Committees of the Hospital Districts of Helsinki and Uusimaa and Southwest Finland, respectively.

### Measures

The FPS and FIREA studies included identical information regarding the variables used in the current study. Work factors included shift work, based on survey responses and categorized in three groups: day work, shift work without night work, shift work with night work. WTC was also based on survey responses and measured with a 7-item Likert scale from 1 (very little) to 5 (very much) regarding the respondent’s ability to influence the following aspects of working hours: duration of work day, beginning and ending times, taking breaks, attending personal affairs, scheduling work shifts, taking vacations/days off, and taking unpaid leave (22, 23). The mean of 7 items was dichotomized at the median (2.60 in FPS and 2.75 in FIREA), thus ‘high WTC’ indicates both intermediate and high scores of WTC. Informal caregiving was assessed with the following question: “Do you provide care to a family member or relative who is unable to take care of himself or herself because of age, illness or disability?” (yes versus no; giving care for own healthy children was not accounted for).

Sleep disturbances were assessed by the Jenkins Sleep Problem Scale (34), which is a self-report scale including the following four types of insomnia symptoms: difficulty in falling asleep, frequent awakenings (ie, difficulty in maintaining sleep), early awakenings and non-restorative sleep (ie, feeling tired and worn out after normal amount of sleep). Participants reported the frequency of sleep disturbances during the past 4 weeks. As previously, the response scale from 1=never to 6=almost every night was dichotomized into no sleep disturbances (0–4 nights/week) and sleep disturbances (5–7 nights/week) for each type of symptoms (22, 35). Any sleep disturbances were indicated as reported having any of the four sleep disturbance types.

Covariates were age, sex, SES, and self-rated health. Age, sex and SES were based on employers’ registers, SES including occupational titles that were coded according to the International Standard Classification of Occupations (ISCO) and categorized into three groups: high (ISCO classes 1–2, eg, teachers, physicians), intermediate (ISCO classes 3–4, eg, registered nurses, technicians), and low (ISCO classes 5–9, eg, cleaners, maintenance workers). Self-rated health was a single-item perception of one’s current health dichotomized into optimal (good, rather good) and non-optimal (average, rather poor, poor) health. Additionally, the following health behaviors were included and they were based on survey responses: height and weight from which body mass index (BMI) was calculated and dichotomized into BMI ≥25 kg/m2 (overweight/obesity) versus less than that; current smoking versus non-smoking; alcohol consumption (weekly average consumption of beer, wine and spirits transformed into grams of alcohol and further dichotomized as risky; >288g among men; >192g among women) (36); and physical activity, which was measured as metabolic equivalent task (MET) (37) hours per week, dichotomized as <14 hours (low physical activity) versus ≥14 hours (moderate/high physical activity).

### Statistical analysis

We used fixed-effect method (38) with conditional logistic regression analysis in which the analyses are performed within individual. This design enables data from longitudinal cohort studies to be used in a case-control design where each individual serves as his/her “case” (disturbed sleep) and “control” (undisturbed sleep). This study design allows individuals to serve as their own control and therefore bias arising from all time invariant factors, such as genetics, response style and personality, is controlled for. To be eligible to the fixed-effect (within-individual) analysis the participants had to respond to at least two study waves. The basic requirement in the fixed-effect method is that the respondent have a change in the outcome (ie, are at one point/study wave cases and another time point controls in relation to sleep disturbance), and there is also variation in the exposure (shift work, WTC, or informal caregiving) in the selected population. The analyses estimate whether working shift work, giving informal care and reporting WTC varied when the participants reported sleep disturbances compared to time(s) when they reported no sleep disturbances.

To estimate a possible dose–response pattern between risk factors and sleep disturbances, we calculated a cumulative exposure variable that described the number of risk factors [0, 1, 2–3 risk factors out of (any form of) shift work, informal caregiving and low WTC].

As a secondary analysis, we undertook repeated measures logistic regression analysis with generalized estimating equations (GEE) method (39) to predict the onset of sleep disturbances among those without sleep disturbances at the baseline survey (a between-individuals approach). The repeated measurements were nested within participants (ie, one individual can contribute to more than once to the prospective design), thus, we used GEE that takes into account the interdependence of observations of the participants that have multiple data points.

Results were presented as odds ratios (OR) with 95% confidence intervals (CI) and the models were adjusted for socio-demographic factors (age, sex, and SES) and health-related factors (overweight/obesity, smoking, risky alcohol use, low physical activity, and self-rated health). In the within-individual fixed-effect analyses, the health-related covariates were used as time-dependent covariates and taken from each measurement point. In the between-individuals analysis with GEE, the covariates were taken from the baseline survey.

We also calculated variables combining the presence or absence of high versus low WTC and other risk factors (any shift work, informal caregiving) to examine whether WTC as a modifiable factor at work plays a role in the potential associations. To examine whether there was statistical interaction between variables, ie, whether there were the joint exposure effects of WTC with shift work or informal caregiving on sleep disturbances, we calculated relative excess risk due to interaction (RERI) which provides a formal assessment of departure from additivity of effects on a relative risk scale (40).

After conducting the analyses separately for FPS and FIREA cohorts, we performed fixed-effect meta-analyses (41) to obtain pooled estimates for the associations. I^2^ statistics was used to test the heterogeneity of results between cohorts. Study-specific analyses were performed with SAS version 9.4 (SAS Institute, Cary, NC, USA) and meta-analyses with Stata 15 (Stata Corp, College Station, TX, USA).

## Results

Descriptive statistics of the participants at their first survey point are presented in supplementary table S1. In FPS, the participants were younger (mean age 55 years) than in FIREA (62.5 years). FIREA participants had in general a better health and sleep profile than FPS participants, but otherwise the cohorts were rather similar with the majority of participants being women (81% in FPS, 80% in FIREA). Of the FPS and FIREA participants, 18.8% and 24.1% of women and 13.7% and 9.0% of men reported shift work with or without night work, respectively, and 16.6% and 17.4% of women and 11.6% and 13.5% of men reported informal caregiving, respectively. Any sleep disturbances were reported by 22.0–30.4% of the participants. The most commonly reported sleep disturbance was frequent awakenings during the night (18.2–25.1%) and the least commonly reported sleep disturbance was difficulty falling asleep (2.5–5.8%). Women reported all types of sleep disturbances more often than men.

**Figure 1:**
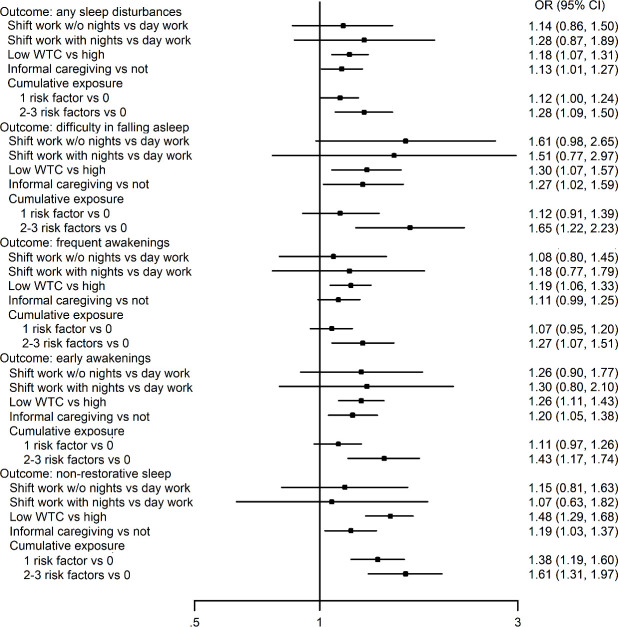
Within-individual associations of shift work, work time control (WTC) and informal care and their accumulation with sleep disturbances; fixed effect conditional logistic regression analysis.

Figure 1 presents results from the pooled within-individual associations between shift work, informal caregiving and WTC, as well as different forms of sleep disturbances. Overall, shift work with or without night work was not associated with any of the examined sleep outcomes. Informal caregiving and low WTC, in turn, were associated with a greater likelihood of all types of sleep disturbances (OR range 1.13–1.48; except the association between informal caregiving and frequent awakenings did not reach statistical significance). When cumulative exposure to 1–≥2 (ie, shift work, low WTC and informal caregiving) was assessed, the findings suggested a relatively consistent dose–response pattern; the greater the number of risk factors, the greater the odds of sleep disturbances (see also supplementary material). Study-specific estimates, number of participants in each analysis and I^2^ heterogeneity indices are presented in supplementary table S2. The I^2^ heterogeneity estimates suggested no significant heterogeneity between FPS and FIREA data.

**Figure 2:**
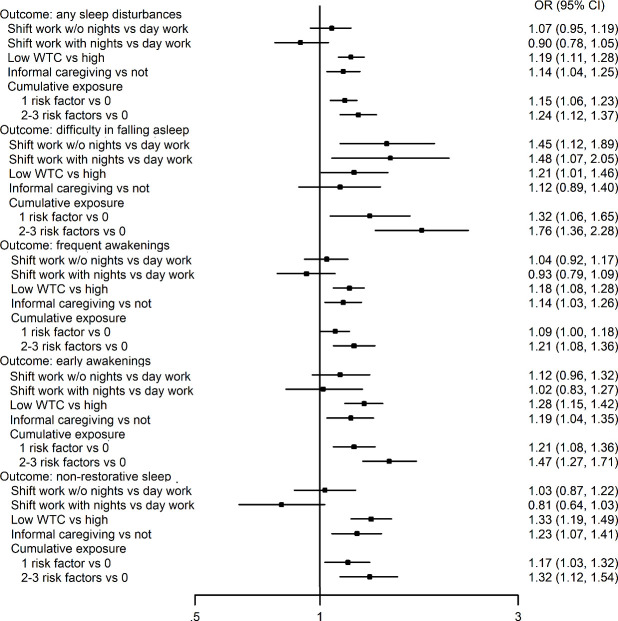
Between-individuals associations of shift work, work time control (WTC) and informal care and their accumulation with onset of sleep disturbances; binary logistic regression analysis adjusted for age, sex and socioeconomic status

The corresponding results from the between-individual analyses are shown in figure 2 and supplementary table S3. Again, shift work was not associated with the onset of sleep disturbances with one exception; shift work with and without night work were both associated with onset of difficulty in falling asleep (OR 1.48 and 1.45, respectively). Otherwise, the results replicated those obtained from the within-individual analyses; low WTC and informal caregiving were relatively consistently associated with all types of sleep disturbances, and, in many outcomes, there was a dose–response pattern with a greater number of risk factors. Of the 29 I^2^ heterogeneity estimates, 6 suggested significant heterogeneity between FPS and FIREA data (supplementary table S3).

**Figure 3:**
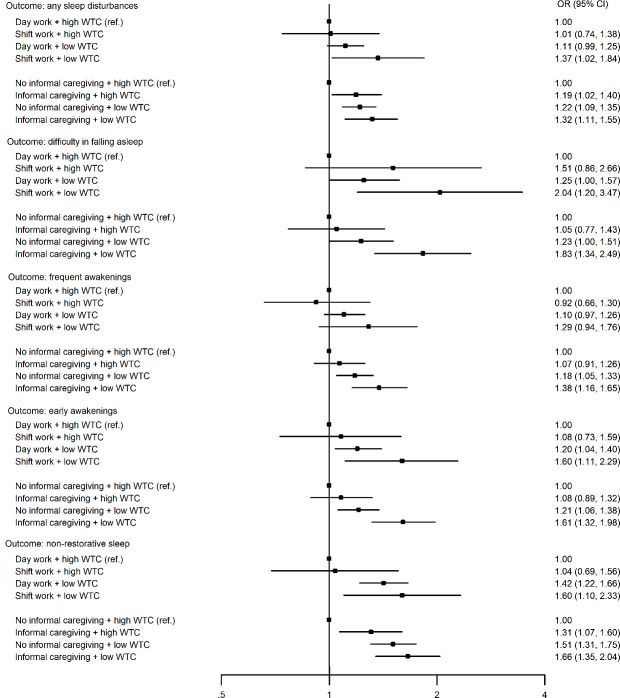
Within-individual associations of shift work and informal care with sleep disturbances by the level of work time control (WTC); fixed effect conditional logistic regression analysis

Results from the within-individual analyses of the association of shift work and informal caregiving with sleep disturbances by the level of WTC are presented in figure 3. Generally, compared with a situation with high WTC and no shift work or informal caregiving, there was a small or non-significant association with sleep disturbances when the participant reported high WTC in combination with shift work or informal caregiving. Respectively, the likelihood of sleep disturbances was greater at times when the participant reported a combination of low WTC and shift work or informal caregiving. However, when we formally tested the interaction by RERI, only two of the RERI estimates were close to statistical significance; the combined association between WTC and informal caregiving predicting difficulty in falling asleep (RERI 0.52, 95% CI -0.004–1.048, P=0.052) and early awakenings (RERI 0.29, 95% CI -0.03–0.61, P=0.076). Study-specific estimates, number of participants in each analysis and I^2^ heterogeneity statistics are presented in supplementary table S4. The I^2^ heterogeneity estimates suggested significant heterogeneity between FPS and FIREA data only in one of the 30 meta-analyses.

**Figure 4:**
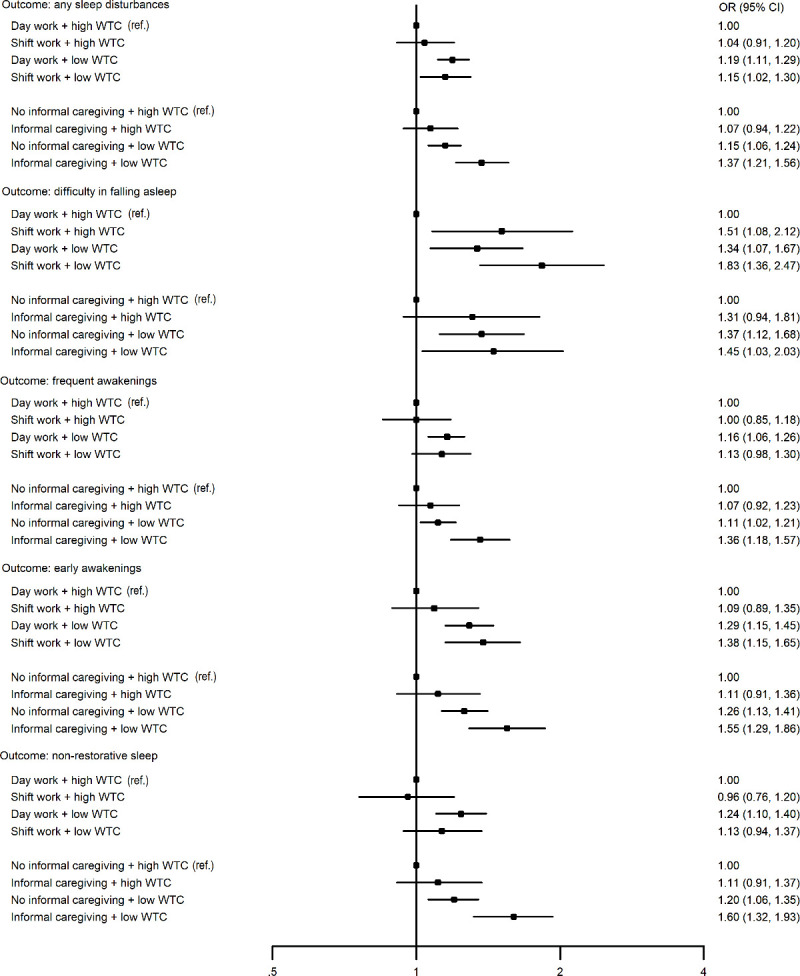
Between-individuals associations of shift work and informal care with onset of sleep disturbances by the level of work time control (WTC); binary logistic regression analysis adjusted for age, sex and socioeconomic status.

Figure 4 shows results from between-individuals analyses on the relation of shift work and informal caregiving with new-onset sleep disturbances by the level of WTC. The findings largely replicate those obtained from within-individual analyses. RERI analyses suggested no departure from additivity. Study-specific data and heterogeneity estimates are presented in supplementary table S5, showing significant heterogeneity in one of the 29 meta-analyses.

Results from the models adjusted for time-dependent health-related covariates (within-individual analyses) and health-related covariates at the baseline survey (between-individuals analyses) are presented in supplementary figures S1–6. There were no major changes in the estimates although they attenuated to some degree.

## Discussion

Results from two cohort studies of older employees showed that low WTC and informal caregiving were associated with various forms of sleep disturbances. We found evidence of a dose–response pattern suggesting a greater likelihood of sleep disturbances with increasing number of the three examined risk factors; shift work, low WTC and informal caregiving. Furthermore, our within-individual analyses showed that informal caregiving was related to a higher risk of sleep disturbances when WTC was low, and to a lower risk when WTC was high.

Shift work alone was generally not associated with sleep disturbances, which is consistent with a recent systematic review of longitudinal studies on shift work and sleep (42). That review found a small non-significant association between shift work and insomnia symptoms (RR 1.16). We found an association between shift work and one symptom of sleep disturbances; compared to day workers, those working in shifts without or with night work had 1.45 and 1.48-fold increased risk of new-onset difficulties in falling asleep in between-individuals analysis. Previous studies suggest that within-individual changes in shiftwork are associated with sleep problems (10), and that a change from non-night to night work increases the risk of common mental disorders (CMD), a correlate of sleep problems, while moving back from night to non-night work increases recovery from CMD (43). However, the study populations in previous studies were different as our cohorts included older employees only. In our study, those who are well-adapted to shift work may continue shift work longer, thus the association between shift work and sleep disturbances may be different among them due to selection. Municipal sector also offers possibilities for employees to transfer from shift to day work, thus our findings are not generalizable to other sectors, especially to those where quitting shift work is not possible. Previous research also suggests acute but not chronic sleep loss among shift workers and that the adverse effects of shift work on sleep may be linked to schedule-related aspects, such as speed of rotation (9). The association found between shift work and difficulty in falling asleep in our study may be linked to various mechanisms such as shift type or length, but also to circadian rhythm disruption, lifestyle changes, or job strain. Because our assessment of shift work only addressed shift work with and without night work and we did not have detailed information on the other shift work characteristics, such as speed of rotation, further research is warranted to examine in greater detail the contribution of different types of shift work.

Our findings on the relation between informal caregiving and sleep disturbances are in agreement with one of the two previous prospective studies which included female long-term care employees characterized as double- or triple-duty (caring for older adults and caring for older adults plus childcare) caregivers because of their occupational roles as nurses (20). In that study, within-individual analysis suggested a weak association with impaired sleep quality for double-duty but not for triple-duty whereas the between-individuals analysis suggested the reverse. In the other longitudinal study with two data points, giving up informal caregiving between baseline and follow-up was associated with reduced risk of sleep disturbances at follow-up (21).

We found an association between low WTC and practically all types of sleep disturbances. In a small longitudinal study of 39 employees, an increase in WTC was associated with improvements in psychomotor vigilance tasks (25). In another study, removal of low WTC could not explain the improved sleep following retirement (35). In our previous prospective study using the FPS data (22), the outcome was onset of sleep disturbances with the total Jenkins score and the used method was a standard between-individuals approach. Here we applied a within-individual approach, examined WTC in combination with shift work and informal caregiving, and analyzed each type of sleep disturbances separately. The mechanisms through which WTC may affect health and well-being include work-non-work balance and job satisfaction although evidence from intervention studies is still sparse (26).

We examined whether low levels of WTC could exacerbate the risk and whether high levels could alleviate the potential adverse impacts of shift work or informal caregiving on sleep disturbances. Although the RERI estimates indicating departure from additivity were not statistically significant, only cautious conclusions can be made of the joint associations. However, the results from the fixed-effect approach give some support to the hypothesis low WTC may increase the risk and high WTC may protect the employee from sleep disturbances when engaged in informal caregiving.

The specific strengths of our study are its large sample size and repeated measurements of exposures and outcomes, which enabled us to apply an advanced analysis method and compare it with a standard prospective approach. The two analytical approaches in our study produced relatively similar findings. We also had the possibility to adjust the models for several time-varying confounders. There were no major changes in the estimates although they somewhat attenuated. Self-rated health was strongly associated with sleep disturbances, thus, adjusting for self-rated health may have been conservative. This is because in addition to poor health status causing sleep disturbances, the direction of association may be bidirectional; ie, poor sleep may also lead to a perception of impaired health (44).

The limitations of our study include that work factors and sleep disturbances were based on self-reports and sleep disturbances were not based on clinically determined sleep disorders. Although the Jenkins scale includes the factor ‘non-restorative sleep’ (ie, feeling tired and worn out after normal amount of sleep), we did not have a measurement of daily functioning due to sleep disturbances. Objective assessment of sleep would be the next step in this field, as in one study where improvements in objective sleep indicators were observed during a year with an improvement of WTC (25). When calculating the cumulative risk score, we used the number of risk factors for each participant as a measure of risk factor burden and did not weight them according to their assumed importance although some of the risk factors may contribute more to sleep disturbances than others. The validity of this assumption should be assessed in future studies. Our assessment of informal caregiving was coarse, therefore future studies are needed to evaluate, for example, the content and impact of tasks in caregiving as well as the difference between child and adult caregiving. A further aspect to which older employees’ control over working hours may have an effect is caring of grandchildren. Regarding WTC, the incongruence between personal need for and access to WTC should be considered in future studies (45). Finally, the female-dominated public sector study population limits the generalizability of our findings to other populations, and we cannot exclude the possibility that some time-varying confounders that were not included (eg, stressful life events) could have biased the results.

In conclusion, this study suggests that informal caregiving and low WTC are associated with risk of sleep disturbances among ageing employees. The findings also suggest that low WTC in combination with informal caregiving may increase the risk of sleep disturbances whereas high WTC may alleviate the adverse impact of informal caregiving on sleep. Intervention studies are needed to confirm whether improving employees’ influence over working hours at the workplace would reduce sleep disturbances and improve quality of sleep.

## Supplementary material

Supplementary material
