# Supplementary material for: Shift work, work time control, and informal caregiving as risk factors for sleep disturbances in an ageing municipal workforce
Source: Scand J Work Environ Health. 2021 Mar 31;47(3):181–90. doi: 10.5271/sjweh.3937 (PMC8126445; doi:10.5271/sjweh.3937)

Shift work, work time control, and informal caregiving as risk factors for sleep disturbances in an ageing municipal workforce [1]

by Marianna Virtanen, PhD,[2] Saana Myllyntausta, PhD, Jenni Ervasti, PhD, Tuula Oksanen, MD, Paula Salo, PhD, Jaana Pentti, BSc, Mika Kivimäki, FMedSci, Annina Ropponen, PhD, Jaana I Halonen, PhD, Jussi Vahtera, MD, Sari Stenholm, PhD

1. *Supplementary material*

2. *Correspondence to: Marianna Virtanen, School of Educational Sciences and Psychology, University of Eastern Finland, FI-80101 Joensuu, Finland. [E-mail: marianna.virtanen@uef.fi]*

Supplementary table S1. Descriptive statistics at the first survey point for fixed-effect analysis in FPS and FIREA studies

	FPS study (n=24,418)		FIREA study (n=2,838)	
	Women (n=19,709)	Men (n=4,709)	Women (n=2,274)	Men (n=564)
Age, mean (SD) years	55.0 (3.6)	55.0 (3.8)	62.4 (1.2)	62.5 (1.3)
Socioeconomic status, n (%): high	7,578 (38.5)	2,248 (47.7)	743 (32.7)	289 (51.2)
intermediate	5,244 (26.6)	1,156 (24.6)	787 (34.6)	104 (18.4)
low	6,887 (34.9)	1,305 (27.7)	744 (32.7)	171 (30.3)
Shift work, n (%): no	15,353 (81.2)	3,684 (86.3)	1,531 (75.9)	430 (91.1)
yes, without nights	2,370 (12.5)	325 (7.6)	296 (14.7)	21 (4.5)
yes, with nights	1,190 (6.3)	262 (6.1)	189 (9.4)	21 (4.5)
Informal caregiving, n (%): no	16,290 (83.4)	4,114 (88.4)	1,849 (82.6)	473 (86.5)
yes	3,242 (16.6)	542 (11.6)	390 (17.4)	74 (13.5)
Work time control (WTC), n (%): high	9,735 (49.8)	2,696 (57.7)	889 (49.4)	287 (61.7)
low	9,822 (50.2)	1,980 (42.3)	909 (50.6)	178 (38.3)
Any sleep disturbances, n (%): no	13,648 (69.6)	3,584 (76.7)	1,618 (71.6)	436 (78.0)
yes	5,966 (30.4)	1,090 (23.3)	642 (28.4)	123 (22.0)
Difficulty in falling asleep, n (%): no	18,327 (94.2)	4,455 (95.8)	2,140 (95.7)	537 (97.5)
yes	1,133 (5.8)	197 (4.2)	97 (4.3)	14 (2.5)
Frequent awakenings, n (%): no	14,575 (75.1)	3,782 (81.8)	1,675 (74.9)	436 (79.7)
yes	4,825 (24.9)	844 (18.2)	562 (25.1)	111 (20.3)
Early awakenings, n (%): no	16,437 (84.6)	4,074 (87.8)	1,948 (88.0)	485 (89.2)
yes	2,992 (15.4)	568 (12.2)	267 (12.1)	59 (10.9)
Feeling tired after normal sleep, n (%): no	16,741 (85.9)	4,148 (89.1)	2,037 (91.2)	521 (95.1)
yes	2,748 (14.1)	506 (10.9)	197 (8.8)	27 (4.9)
Physical activity, n (%): intermediate/high	12,161 (62.0)	2,986 (64.0)	1,420 (63.0)	355 (64.1)
low	7,443 (38.0)	1,678 (36.0)	835 (37.0)	199 (35.9)
Overweight/obesity, n (%): no	8,906 (47.5)	1,457 (32.2)	939 (42.2)	165 (29.9)
yes	9,828 (52.5)	3,064 (67.8)	1,288 (57.8)	387 (70.1)
Smoking, n (%): no	16,693 (86.5)	3,853 (83.4)	2,013 (90.8)	497 (89.4)
yes	2,617 (13.6)	768 (16.6)	205 (9.2)	59 (10.6)
Risky alcohol use, n (%): no	17,965 (91.6)	4,248 (91.1)	2,073 (91.6)	491 (87.8)
yes	1,644 (8.4)	413 (8.9)	190 (8.4)	68 (12.2)
Self-rated health, n (%): optimal	13,815 (70.6)	3,260 (69.8)	1,808 (79.7)	427 (76.8)
non-optimal	5,757 (29.4)	1,413 (30.2)	460 (20.3)	129 (23.2)

Supplementary table S2. Study-specific results from within-individual analyses of the association of shift work, work time control (WTC) and informal caregiving with sleep disturbances, and heterogeneity estimates from meta-analyses pooling FPS and FIREA data

	FPS study			FIREA study			Meta-analysis	
	OR	(95% CI)	n	OR	95% CI	n	I^2 (%)	P value
Outcome: any sleep disturbances								
Shift work without nights vs day work	1.13	(0.85-1.50)	14679	1.29	(0.36- 4.58)	947	0.0	0.842
Shift work with nights vs day work	1.30	(0.87-1.93)	14679	1.08	(0.25- 4.74)	947	0.0	0.812
Low WTC vs high	1.19	(1.07-1.32)	15667	1.04	(0.68- 1.60)	937	0.0	0.549
Informal caregiving vs not	1.15	(1.02-1.29)	15578	0.92	(0.60- 1.42)	1562	0.0	0.327
Cumulative exposure								
1 risk factor vs 0	1.12	(1.00-1.25)	14400	1.04	(0.68- 1.60)	872	0.0	0.743
2-3 risk factors vs 0	1.30	(1.10-1.53)	14400	0.90	(0.42- 1.93)	872	0.0	0.356
Outcome: difficulty in falling asleep								
Shift work without nights vs day work	1.58	(0.95-2.63)	4031	2.34	(0.26- 21.41)	195	0.0	0.734
Shift work with nights vs day work	1.62	(0.80-3.27)	4031	0.71	(0.07- 7.54)	195	0.0	0.508
Low WTC vs high	1.29	(1.06-1.56)	4339	1.46	(0.53- 4.05)	206	0.0	0.815
Informal caregiving vs not	1.28	(1.02-1.60)	4306	1.14	(0.40- 3.26)	317	0.0	0.832
Cumulative exposure								
1 risk factor vs 0	1.13	(0.91-1.41)	3931	0.96	(0.29- 3.18)	182	0.0	0.793
2-3 risk factors vs 0	1.66	(1.22-2.26)	3931	1.40	(0.28- 7.02)	182	0.0	0.839
Outcome: frequent awakenings								
Shift work without nights vs day work	1.07	(0.79-1.45)	12627	1.25	(0.33- 4.69)	871	0.0	0.823
Shift work with nights vs day work	1.21	(0.78-1.88)	12627	0.84	(0.19- 3.66)	871	0.0	0.643
Low WTC vs high	1.22	(1.09-1.37)	13462	0.79	(0.50- 1.24)	851	69.8	0.069
Informal caregiving vs not	1.13	(1.00-1.28)	13399	0.92	(0.59- 1.44)	1467	0.0	0.384
Cumulative exposure								
1 risk factor vs 0	1.09	(0.96-1.23)	12392	0.81	(0.52- 1.26)	803	37.6	0.205
2-3 risk factors vs 0	1.31	(1.09-1.56)	12392	0.67	(0.30- 1.51)	803	60.3	0.112
Outcome: early awakenings								
Shift work without nights vs day work	1.28	(0.91-1.82)	9513	0.85	(0.15- 4.74)	500	0.0	0.649
Shift work with nights vs day work	1.34	(0.82-2.22)	9513	0.84	(0.13- 5.27)	500	0.0	0.633
Low WTC vs high	1.27	(1.12-1.45)	10208	1.05	(0.56- 1.94)	501	0.0	0.557
Informal caregiving vs not	1.20	(1.04-1.38)	10154	1.28	(0.72- 2.27)	809	0.0	0.831
Cumulative exposure								
1 risk factor vs 0	1.12	(0.98-1.29)	9334	0.85	(0.46- 1.57)	466	0.0	0.390
2-3 risk factors vs 0	1.44	(1.18-1.76)	9334	1.08	(0.38- 3.05)	466	0.0	0.595
Outcome: non-restorative sleep								
Shift work without nights vs day work	1.19	(0.84-1.70)	8713	0.24	(0.02- 2.35)	343	41.1	0.193
Shift work with nights vs day work	1.16	(0.68-2.00)	8713	0.12	(0.01- 2.71)	343	58.8	0.119
Low WTC vs high	1.47	(1.29-1.69)	9386	1.62	(0.77- 3.40)	354	0.0	0.801
Informal caregiving vs not	1.18	(1.02-1.36)	9332	1.54	(0.72- 3.29)	548	0.0	0.500
Cumulative exposure								
1 risk factor vs 0	1.37	(1.18-1.60)	8567	1.72	(0.79- 3.72)	322	0.0	0.572
2-3 risk factors vs 0	1.60	(1.30-1.97)	8567	1.94	(0.55- 6.91)	322	0.0	0.768

Supplementary table S3. Study-specific results from between-individuals analyses of the association of shift work, work time control (WTC) and informal caregiving with sleep disturbances, and heterogeneity estimates from meta-analyses pooling FPS and FIREA data

	FPS study						FIREA study						Meta-analysis	
	OR[a]	(95% CI)[a]	$N_{exposed}$ cases	$N_{exposed}$ all	$N_{exposed}$ cases	$N_{exposed}$ all	OR[a]	95% CI[a]	$N_{exposed}$ cases	$N_{exposed}$ all	$N_{exposed}$ cases	$N_{exposed}$ all	I^2 (%)	P value
Outcome: any sleep disturbances														
Shift work without nights vs day work	1.01	(0.90- 1.14)	435	2355	3070	17921	1.73	(1.21-2.47)	50	270	206	1787	87.3	0.005
Shift work with nights vs day work	0.93	(0.80- 1.08)	231	1339	3070	17921	0.58	(0.32-1.05)	13	183	206	1787	56.2	0.131
Low WTC vs average/high	1.20	(1.12- 1.30)	1310	6799	2583	15761	1.04	(0.79-1.37)	114	934	135	1178	0.0	0.325
Informal caregiving vs not	1.16	(1.05- 1.27)	664	3429	3240	19095	0.93	(0.67-1.29)	49	429	286	2375	38.0	0.204
Cumulative exposure														
1 risk factor vs 0	1.16	(1.07- 1.25)	1699	9472	1295	8339	1.00	(0.77-1.28)	117	992	171	1456	16.5	0.274
2-3 risk factors vs 0	1.25	(1.12- 1.39)	695	3554	1295	8339	1.14	(0.81-1.61)	52	390	171	1456	0.0	0.616
P-value for trend	<0.0001						0.55							
Outcome: difficulty in falling asleep														
Shift work without nights vs day work	1.41	(1.08- 1.85)	74	2335	348	17802	2.30	(0.79-6.69)	5	263	13	1770	0.0	0.384
Shift work with nights vs day work	1.49	(1.07- 2.08)	45	1333	348	17802	1.23	(0.23-6.61)	2	182	13	1770	0.0	0.826
Low WTC vs average/high	1.25	(1.03- 1.51)	174	6751	323	15659	0.53	(0.20-1.45)	7	919	13	1171	64.0	0.096
Informal caregiving vs not	1.14	(0.90- 1.44)	86	3407	414	18968	0.73	(0.26-2.07)	4	428	28	2343	0.0	0.411
Cumulative exposure														
1 risk factor vs 0	1.42	(1.13- 1.79)	216	9398	130	8295	0.52	(0.23-1.18)	8	976	19	1442	81.4	0.020
2-3 risk factors vs 0	1.87	(1.43- 2.45)	114	3531	130	8295	0.69	(0.24-1.98)	5	386	19	1442	68.9	0.073
P-value for trend	<0.0001						0.29							
Outcome: frequent awakenings														
Shift work without nights vs day work	0.97	(0.85- 1.10)	318	2327	2262	17694	1.83	(1.25-2.69)	42	267	166	1766	89.4	0.002
Shift work with nights vs day work	0.95	(0.80- 1.12)	178	1323	2262	17694	0.67	(0.36-1.26)	12	180	166	1766	10.2	0.291
Low WTC vs high	1.19	(1.09- 1.30)	965	6701	1905	15575	1.05	(0.77-1.42)	92	920	109	1164	0.0	0.441
Informal caregiving vs not	1.16	(1.05- 1.29)	497	3395	2381	18844	0.89	(0.62-1.28)	39	427	235	2343	47.4	0.168
Cumulative exposure														
1 risk factor vs 0	1.10	(1.01- 1.21)	1240	9363	965	8227	0.95	(0.72-1.26)	92	986	141	1435	0.0	0.328
2-3 risk factors vs 0	1.21	(1.08- 1.37)	524	3511	965	8227	1.21	(0.84-1.75)	44	382	141	1435	0.0	1.000
P-value for trend	0.001						0.52							

Table continues

Table continued

Outcome: early awakenings														
Shift work without nights vs day work	1.06	(0.90- 1.25)	184	2323	1257	17766	2.45	(1.35-4.45)	18	266	51	1767	85.8	0.008
Shift work with nights vs day work	1.03	(0.83- 1.28)	103	1320	1257	17766	0.90	(0.34-2.42)	5	180	51	1767	0.0	0.792
Low WTC vs high	1.28	(1.15- 1.43)	572	6730	1038	15615	1.17	(0.71-1.92)	35	917	34	1168	0.0	0.729
Informal caregiving vs not	1.19	(1.04- 1.36)	284	3398	1333	18913	1.14	(0.64-2.01)	17	429	81	2334	0.0	0.886
Cumulative exposure														
1 risk factor vs 0	1.22	(1.08- 1.37)	700	9385	503	8272	1.11	(0.69-1.79)	35	980	45	1433	0.0	0.706
2-3 risk factors vs 0	1.47	(1.26- 1.71)	315	3507	503	8272	1.49	(0.84-2.62)	19	384	45	1433	0.0	0.964
P-value for trend	<0.0001						0.21							
Outcome: non-restorative sleep														
Shift work without nights vs day work	0.99	(0.83- 1.18)	166	2337	1155	17819	2.10	(1.01-4.33)	11	267	37	1775	74.2	0.049
Shift work with nights vs day work	0.81	(0.64- 1.03)	80	1329	1155	17819	n.a.	n.a.	18	924	30	1170	n.a.	n.a.
Low WTC vs high	1.36	(1.21- 1.52)	535	6756	933	15667	0.72	(0.39-1.33)	7	427	52	2349	74.9	0.046
Informal caregiving vs not	1.25	(1.09- 1.44)	269	3415	1198	18974	0.73	(0.33-1.60)					42.2	0.188
Cumulative exposure														
1 risk factor vs 0	1.18	(1.04- 1.34)	635	9420	466	8289	0.98	(0.57-1.69)	21	983	32	1441	0.0	0.514
2-3 risk factors vs 0	1.34	(1.14- 1.58)	278	3528	466	8289	0.82	(0.36-1.87)	7	386	32	1441	23.9	0.252
P-value for trend	0.0002						0.68							

[a]Adjusted for age, sex and socioeconomic status.

Note. $N_{exposed\ cases}$ is the number of cases among the exposed group and $N_{exposed\ all}$ is the total number of participants among the exposed group.

Supplementary table S4. Study-specific results from within-individual analyses of the association of shift work and informal caregiving with sleep disturbances by the level of work time control (WTC), and heterogeneity estimates from meta-analyses pooling FPS and FIREA data

	FPS study				FIREA study				Meta-analysis	
	OR	95% CI	n of cases	n	OR	95% CI	n of cases	n	I^2 (%)	P value
Outcome: any sleep disturbances										
Day work + high WTC (ref.)	1.00		2945	6160	1.00		208	446		
Shift work + high WTC	1.02	(0.74-1.40)	466	997	0.90	(0.24- 3.41)	24	54	0.0	0.857
Day work + low WTC	1.11	(0.98-1.25)	2932	5944	1.10	(0.67- 1.81)	122	258	0.0	0.972
Shift work + low WTC	1.38	(1.02-1.87)	757	1509	1.19	(0.34- 4.19)	67	136	0.0	0.822
No informal caregiving + high WTC (ref.)	1.00		2903	6190	1.00		207	438		
Informal caregiving + high WTC	1.22	(1.03-1.43)	654	1307	0.80	(0.41- 1.54)	33	76	32.1	0.225
No informal caregiving + low WTC	1.23	(1.10-1.37)	3186	6455	1.02	(0.65- 1.60)	160	340	0.0	0.429
Informal caregiving + low WTC	1.32	(1.12-1.57)	747	1486	1.18	(0.50- 2.81)	31	59	0.0	0.803
Outcome: difficulty in falling asleep										
Day work + high WTC (ref.)	1.00		660	1456	1.00		36	76		
Shift work + high WTC	1.55	(0.87- 2.78)	134	304	0.88	(0.07- 11.87)	7	16	0.0	0.673
Day work + low WTC	1.25	(0.99-1.57)	766	1656	1.33	(0.37- 4.76)	31	66	0.0	0.925
Shift work + low WTC	2.08	(1.21- 3.60)	284	582	1.48	(0.15- 14.66)	15	33	0.0	0.777
No informal caregiving + high WTC (ref.)	1.00		659	1478	1.00		37	81		
Informal caregiving + high WTC	1.07	(0.78-1.46)	179	389	0.54	(0.09- 3.40)	4	11	0.0	0.467
No informal caregiving + low WTC	1.23	(1.00-1.52)	873	1886	1.20	(0.37- 3.87	38	86	0.0	0.968
Informal caregiving + low WTC	1.80	(1.32- 2.47)	249	501	2.95	(0.46- 18.99)	11	19	0.0	0.608
Outcome: frequent awakenings										
Day work + high WTC (ref.)	1.00		2480	5248	1.00		190	401		
Shift work + high WTC	0.92	(0.65-1.31)	382	850	0.99	(0.26- 3.73)	28	59	0.0	0.917
Day work + low WTC	1.12	(0.98-1.28)	2513	5122	0.86	(0.50- 1.47)	104	230	0.0	0.351
Shift work + low WTC	1.32	(0.96-1.83)	679	1341	0.89	(0.25- 3.12)	63	127	0.0	0.553
No informal caregiving + high WTC (ref.)	1.00		2446	5257	1.00		197	405		
Informal caregiving + high WTC	1.10	(0.92-1.30)	546	1147	0.70	(0.36- 1.38)	30	70	38.7	0.202
No informal caregiving + low WTC	1.22	(1.08-1.38)	2709	5524	0.74	(0.46- 1.19)	142	313	74.9	0.046
Informal caregiving + low WTC	1.41	(1.18-1.69)	692	1343	0.75	(0.28- 1.98)	25	49	35.4	0.213
Outcome: early awakenings										
Day work + high WTC (ref.)	1.00		1743	3825	1.00		108	227		
Shift work + high WTC	1.15	(0.77-1.71)	289	643	0.32	(0.06- 1.86)	11	29	50.6	0.155
Day work + low WTC	1.22	(1.05-1.42)	1887	3912	0.88	(0.42- 1.84)	69	147	0.0	0.396
Shift work + low WTC	1.66	(1.14- 2.40)	532	1081	0.74	(0.14- 3.75)	32	70	0.0	0.347
No informal caregiving + high WTC (ref.)	1.00		1737	3871	1.00		102	218		
Informal caregiving + high WTC	1.09	(0.89-1.33)	396	839	0.96	(0.37- 2.46)	18	43	0.0	0.797
No informal caregiving + low WTC	1.22	(1.07-1.40)	2052	4286	1.00	(0.53- 1.91)	81	186	0.0	0.552
Informal caregiving + low WTC	1.59	(1.29-1.95)	541	1071	2.45	(0.78- 7.68)	25	44	0.0	0.466
Outcome: non-restorative sleep										
Day work + high WTC (ref.)	1.00		1589	3496	1.00		67	153		
Shift work + high WTC	1.09	(0.72-1.65)	230	523	0.20	(0.02- 2.30)	5	17	47.5	0.168
Day work + low WTC	1.41	(1.20-1.65)	1791	3665	1.81	(0.75- 4.35)	56	114	0.0	0.584
Shift work + low WTC	1.65	(1.13- 2.41)	492	992	0.47	(0.04- 5.32)	19	44	0.0	0.320
No informal caregiving + high WTC (ref.)	1.00		1518	3446	1.00		65	155		
Informal caregiving + high WTC	1.29	(1.06-1.59)	394	802	2.69	(0.64- 11.42)	10	20	0.0	0.322
No informal caregiving + low WTC	1.51	(1.31-1.75)	1937	3977	1.61	(0.74- 3.50)	68	148	0.0	0.874
Informal caregiving + low WTC	1.63	(1.32- 2.01)	522	1034	3.34	(0.82- 13.57)	12	22	0.0	0.322

Supplementary table S5. Study-specific results from between-individuals analyses of the association of shift work and informal caregiving with sleep disturbances by the level of work time control (WTC), and heterogeneity estimates from meta-analyses pooling FPS and FIREA data

	FPS study				FIREA study				Meta-analysis	
Outcome: any sleep disturbances	OR[a]	95% CI[a]	n of cases	n	OR[a]	95% CI[a]	n of cases	n	I^2 (%)	P value
Day work + high WTC	1.00		1549	9861	1.00		117	1011		
Shift work + high WTC	1.04	(0.90-1.20)	276	1596	1.12	(0.60- 2.09)	14	116	0.0	0.821
Day work + low WTC	1.21	(1.12-1.31)	1503	7996	0.94	(0.68- 1.31)	69	624	53.6	0.142
Shift work + low WTC	1.13	(0.99-1.28)	386	2084	1.45	(0.94- 2.24)	39	245	14.2	0.280
No informal caregiving + high WTC	1.00		1594	10040	1.00		112	1001		
Informal caregiving + high WTC	1.07	(0.93-1.22)	301	1767	1.10	(0.66- 1.85)	20	163	0.0	0.919
No informal caregiving + low WTC	1.15	(1.06-1.24)	1618	8934	1.10	(0.82- 1.49)	97	767	0.0	0.778
Informal caregiving + low WTC	1.41	(1.24-1.60)	355	1640	0.81	(0.46- 1.45)	15	154	70.7	0.065
Outcome: difficulty in falling asleep										
Day work + high WTC	1.00		162	9810	1.00		11	1006		
Shift work + high WTC	1.55	(1.10-2.19)	46	1582	0.63	(0.08- 4.89)	1	115	0.0	0.397
Day work + low WTC	1.38	(1.11-1.73)	183	7930	0.25	(0.05- 1.29)	2	613	76.0	0.041
Shift work + low WTC	1.86	(1.37-2.52)	72	2072	1.32	(0.33- 5.30)	5	241	0.0	0.636
No informal caregiving + high WTC	1.00		179	9984	1.00		10	994		
Informal caregiving + high WTC	1.28	(0.91-1.80)	41	1756	1.71	(0.48- 6.15)	3	163	0.0	0.667
No informal caregiving + low WTC	1.41	(1.15-1.74)	230	8863	0.72	(0.26- 1.99)	7	753	37.9	0.205
Informal caregiving + low WTC	1.45	(1.03-2.03)	44	1630	n.a.	n.a. n.a.	0	154	na	na
Outcome: frequent awakenings										
Day work + high WTC	1.00		1152	9737	1.00		94	998		
Shift work + high WTC	0.98	(0.83-1.16)	203	1583	1.39	(0.73- 2.66)	13	116	5.0	0.305
Day work + low WTC	1.17	(1.07-1.28)	1099	7896	0.97	(0.68- 1.39)	55	616	0.0	0.319
Shift work + low WTC	1.09	(0.94-1.26	290	2054	1.61	(1.01- 2.58)	33	239	58.7	0.120
No informal caregiving + high WTC	1.00		1182	9908	1.00		90	987		
Informal caregiving + high WTC	1.06	(0.91-1.23)	224	1753	1.14	(0.65- 2.01)	17	163	0.0	0.807
No informal caregiving + low WTC	1.11	(1.01-1.21)	1181	8819	1.11	(0.80- 1.54)	78	756	0.0	1.000
Informal caregiving + low WTC	1.39	(1.20-1.61)	268	1620	0.88	(0.47- 1.63)	13	152	49.1	0.161
Outcome: early awakenings										
Day work + high WTC	1.00		605	9780	1.00		28	1002		
Shift work + high WTC	1.06	(0.86-1.32)	109	1579	2.12	(0.79- 5.69)	6	116	44.7	0.179
Day work + low WTC	1.30	(1.15-1.46)	639	7924	1.07	(0.58- 1.98)	18	613	0.0	0.542
Shift work + low WTC	1.34	(1.12-1.62)	176	2050	2.11	(1.02- 4.38)	14	239	28.7	0.236
No informal caregiving + high WTC	1.00		622	9953	1.00		27	991		
Informal caregiving + high WTC	1.10	(0.89-1.34)	120	1750	1.40	(0.56- 3.52)	6	163	0.0	0.616
No informal caregiving + low WTC	1.26	(1.12-1.41)	699	8840	1.33	(0.78- 2.27)	30	750	0.0	0.846
Informal caregiving + low WTC	1.57	(1.30-1.89)	158	1626	1.04	(0.39- 2.79)	5	154	0.0	0.420
Outcome: non-restorative sleep										
Day work + high WTC	1.00		558	9773	1.00		25	1005		
Shift work + high WTC	0.94	(0.74-1.18)	123	1953	1.40	(0.44- 4.53)	4	115	0.0	0.511
Day work + low WTC	1.26	(1.12-1.43)	590	7982	0.69	(0.32- 1.47)	11	618	57.2	0.126
Shift work + low WTC	1.15	(0.95-1.40)	190	2582	0.78	(0.29- 2.13)	5	242	0.0	0.454
No informal caregiving + high WTC	1.00		570	9981	1.00		26	993		
Informal caregiving + high WTC	1.12	(0.91-1.38)	113	1760	0.95	(0.34- 2.66)	4	163	0.0	0.758
No informal caregiving + low WTC	1.22	(1.08-1.38)	620	8872	0.72	(0.37- 1.39)	15	758	57.6	0.125
Informal caregiving + low WTC	1.63	(1.35-1.97)	151	1633	0.45	(0.10- 1.96)	2	153	64.7	0.093

[a]Adjusted for age, sex and socioeconomic status.

Supplementary figure S1. Within-individual associations of shift work, work time control (WTC) and informal care and their accumulation with sleep disturbances; fixed effect conditional logistic regression analysis adjusted for time-dependent health-related covariates

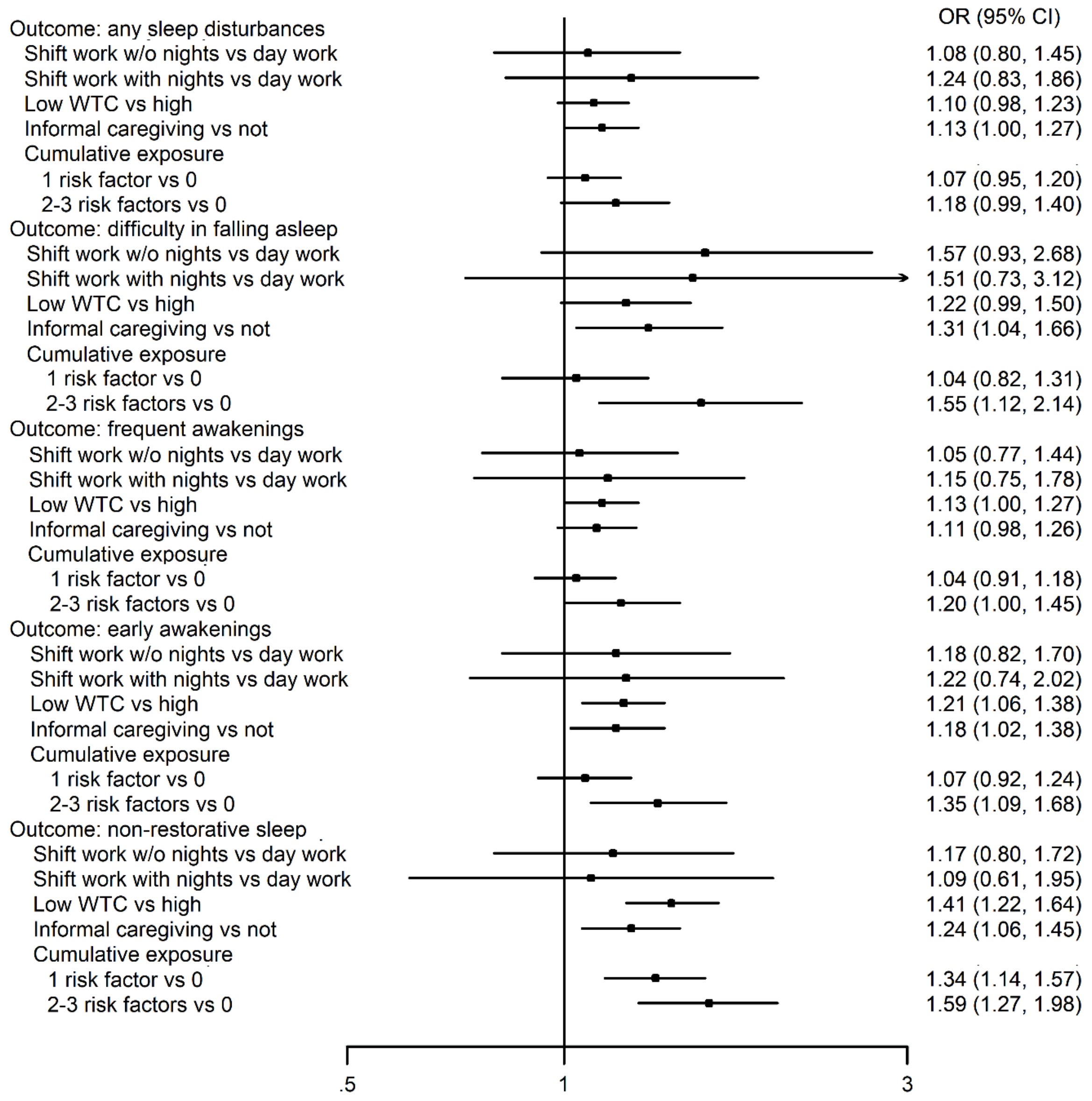

Supplementary figure S2. Between-individuals associations of shift work, work time control (WTC) and informal care and their accumulation with onset of sleep disturbances; binary logistic regression analysis adjusted for age, sex, socioeconomic status and health-related covariates at baseline

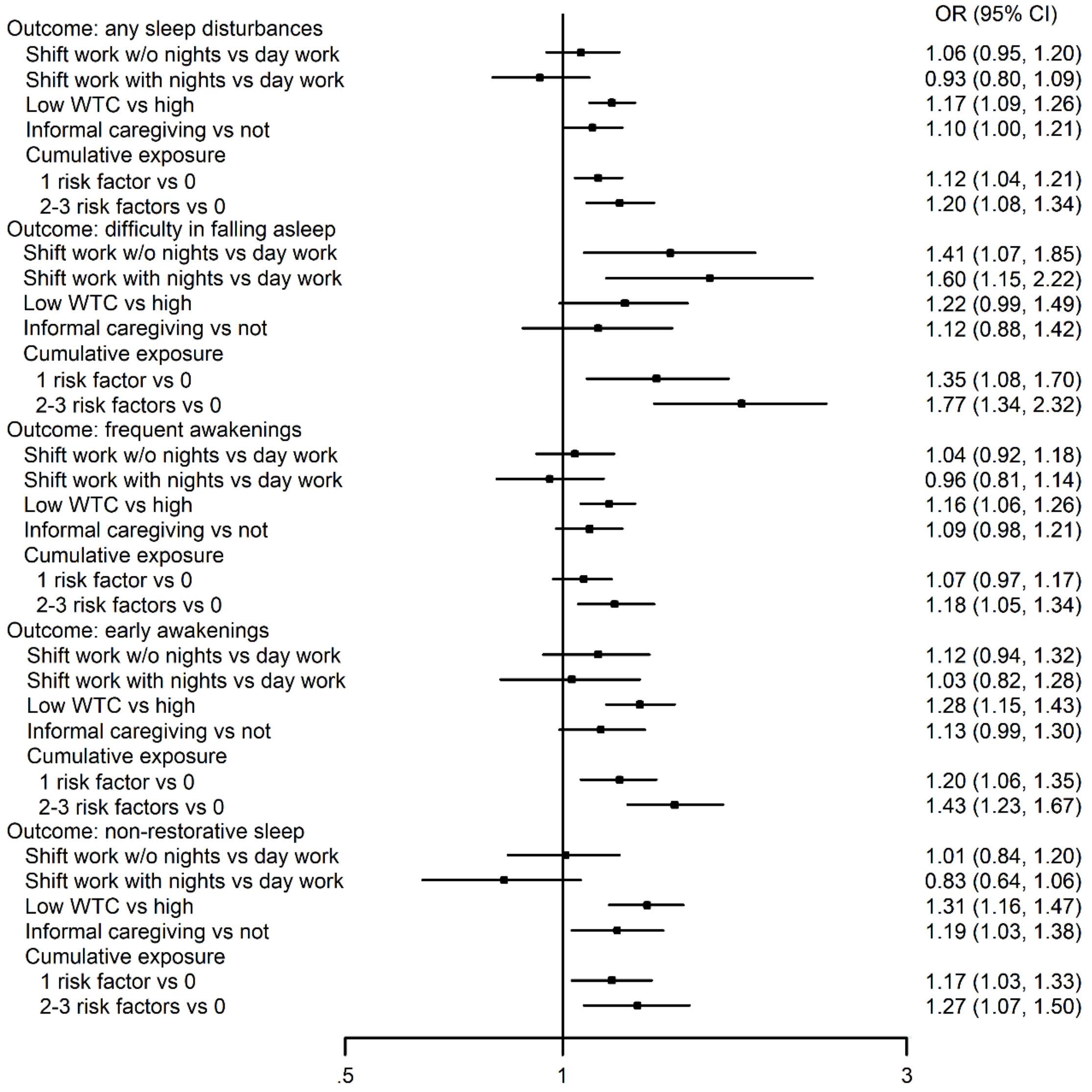

Supplementary figure S3. Within-individual associations of shift work and informal care with sleep disturbances by the level of work time control (WTC); fixed effect conditional logistic regression analysis adjusted for time-dependent health-related covariates

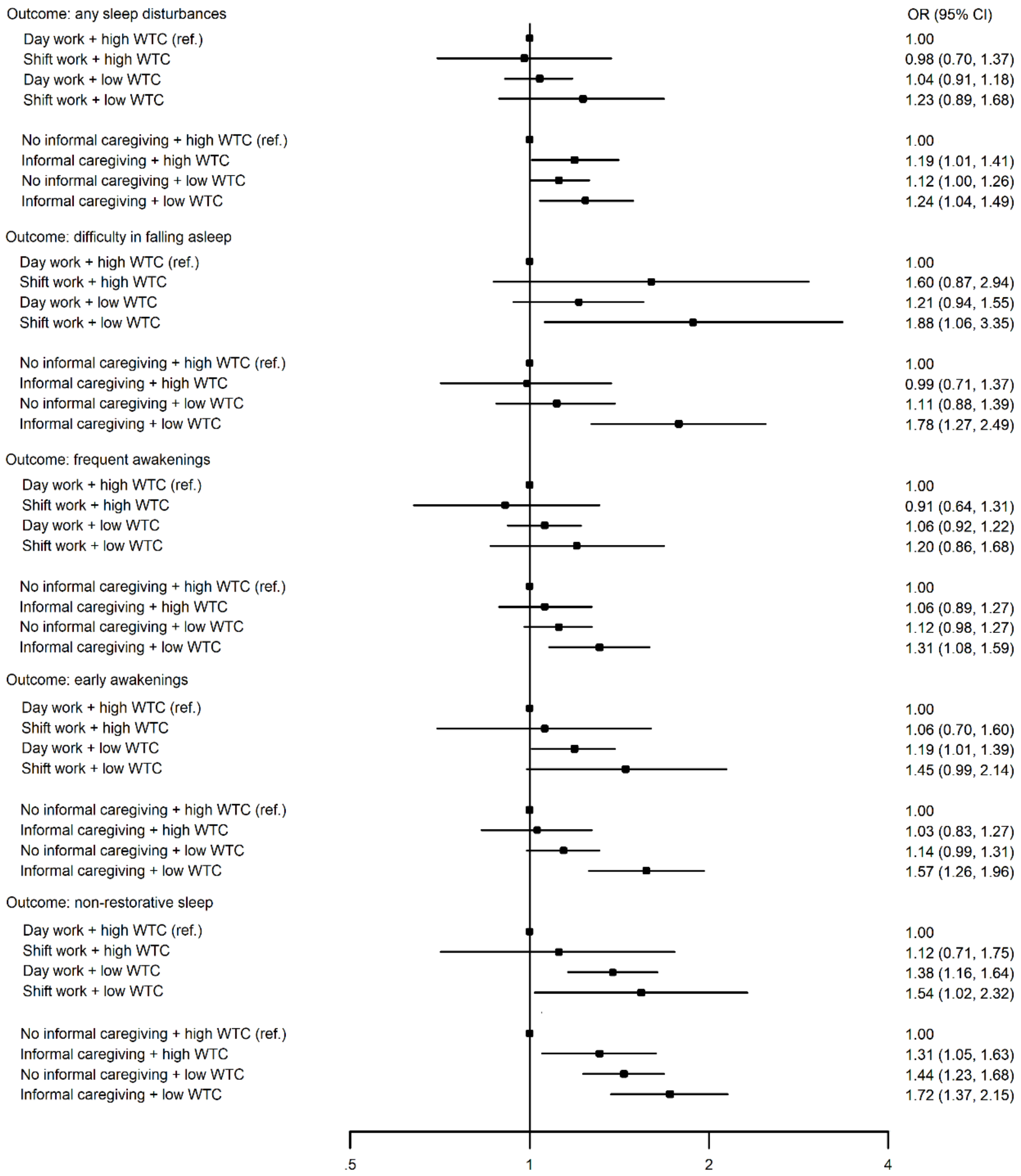

Supplementary figure S4. Between-individuals associations of shift work and informal care with sleep disturbances by the level of work time control (WTC); binary logistic regression analysis adjusted for age, sex, socioeconomic status and health-related covariates

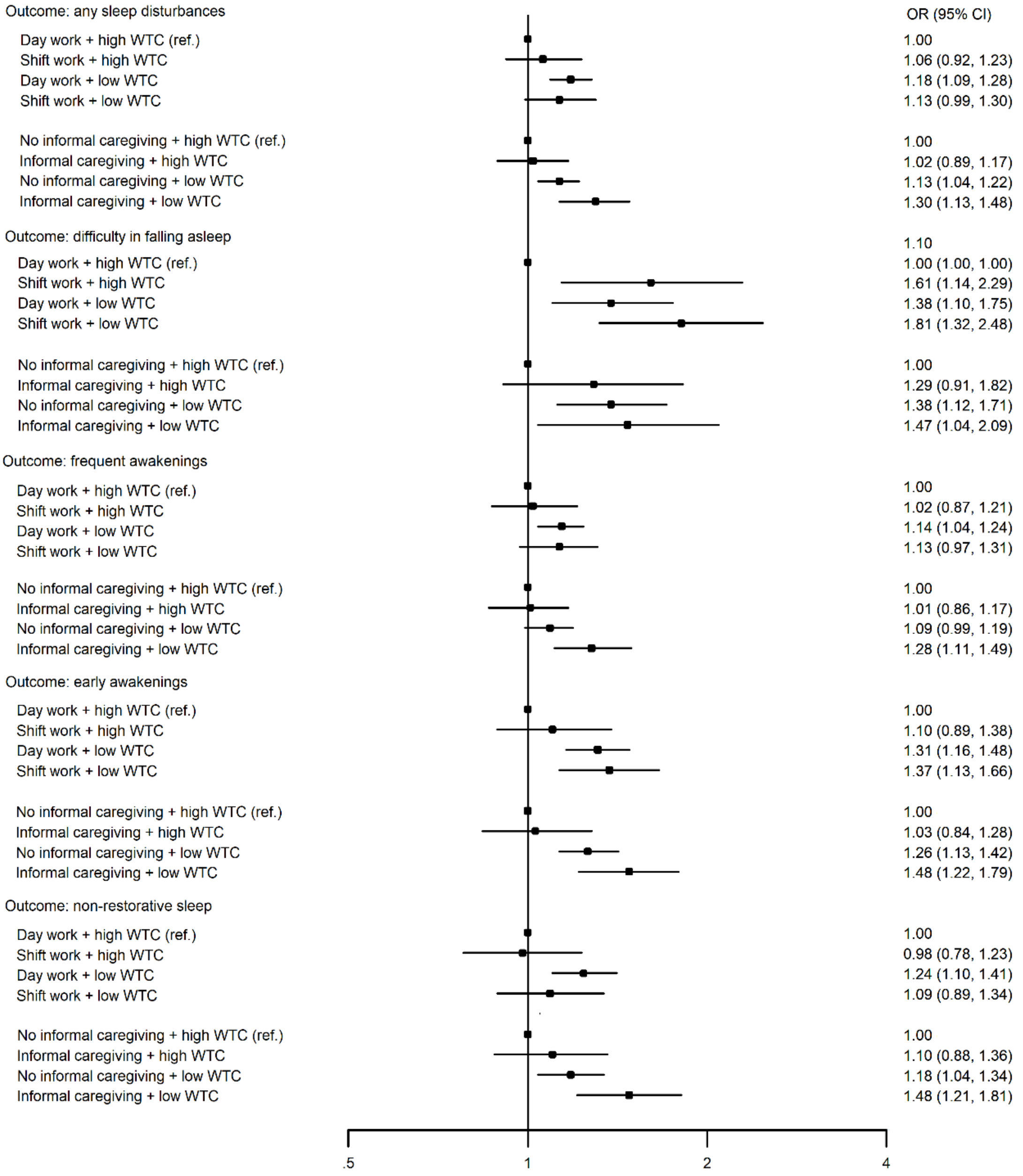

Supplement: Supplementary material [file SJWEH-47-181-S001.pdf]
